# The Experiences of Grief and Personal Growth in University Students: A Qualitative Study

**DOI:** 10.3390/ijerph18041899

**Published:** 2021-02-16

**Authors:** Jovita Tan, Karl Andriessen

**Affiliations:** Centre for Mental Health, Melbourne School of Population and Global Health, The University of Melbourne, Parkville, VIC 3010, Australia; jovitat@student.unimelb.edu.au

**Keywords:** grief, bereavement, mental health, social support, help-seeking, students, young adults, personal growth, posttraumatic growth

## Abstract

Background: Experiencing the death of a close person, especially in emerging adults and students, can have profound effects on the bereaved individual’s life. As most research in this field has focused on negative effects of a loss, little is known about potential positive effects experienced by bereaved university students. This study investigated the experience of grief and personal growth in a sample of students from The University of Melbourne, Australia. Methods: Semi-structured interviews via Zoom/telephone with bereaved students (*n* = 14), who were invited to reflect on their loss and any personal growth potentially experienced. Thematic analysis of the data was based on a deductive and inductive approach. Results: The analysis identified four themes: (i) sharing of grief as a coping mechanism, (ii) balance between grief reactions and moving forward in life, (iii) lessons learned and personal growth, and (iv) adopting values from the deceased person and continuing bonds. Conclusions: Participants emphasized personal growth regarding self-perception and philosophical views on life. Following the loss, they preferred peer support, and used formal services only when they had a specific need. The findings indicate the importance of social support for bereaved students, and the complimentary role of peer and professional support. Hence, academic institutions should offer supportive services tailored to both students and professionals to help bereaved students.

## 1. Introduction

All people face the inevitability of death amongst close friends and family. Particularly in college and university students, 40% of students have experienced the death of a friend or relative before the age of 18 [1]. Common grief reactions include emotions such as sadness, anger and longing for the deceased person. Grief in university students may also entail physical and mental distress, insomnia, and loss of motivation [2].

The university constitutes a new environment for emerging adults (aged 18–30 years) as it represents a period in life where they transition from a ‘dependent’ high school environment to one that is more independent, akin to adult life. Independence and maturity amongst emerging adults may be developed in regard to gaining independence from home, identity formation, developing and strengthening interpersonal relationships and moral reasoning [1,3].

However, during this period of rapid transitions, emerging adults, including students, are also vulnerable to mental health problems. These comprise a major contribution to the burden of disease in this population group as 50% to 75% of mental health problems in adulthood have their onset before the age of 24 years [4,5,6]. Experiencing grief and bereavement may cause extra distress and contribute to mental health issues, such as anxiety, depression and substance abuse [7,8]. Especially in students, it may affect academic performance and quality of life [2]. However, research over the last two decades indicates that grief can also have positive outcomes regarding personal growth experienced by the bereaved individual [9].

The concept of personal growth is derived from posttraumatic growth (PTG) [9]. Personal growth is understood as perceiving positive outcomes in various aspects of life following the loss of a significant individual. It may refer to a bereaved individual’s ability to overcome challenges through a change in their personality such as developing hardiness or through a change in their relationships with others [10,11]. Personal growth has been further identified as an outcome arising when the loss challenges an individual’s perception of the world. Similar to PTG, it comprises the following five domains: (1) self-perception, (2) increased sense of closeness with others, (3) new possibilities, (4) appreciation for life and (5) spiritual/existential changes [12]. During adolescence, personal growth specifically entails experiencing changes in self-perception, valuing relationships and life, or learning ‘life lessons’ [13]. Likewise, within students and young adults, emotional closeness with others and positive help-seeking attitudes are associated with experiencing personal growth [14,15]. Psychosocial and grief interventions can facilitate personal growth in bereaved students, and personal growth may occur after any cause of either expected or unexpected death [16,17].

### 1.1. Formal and Informal Help-Seeking

Coping mechanisms typically adopted by bereaved students comprise a mixture of formal and informal methods [18,19,20]. Formal methods are defined as those involving the use of professional counselling or psychological services [21]. Australian universities commonly offer these services for free to all students. However, they are usually in high demand, presenting a barrier for those who require the help [22]. Furthermore, some of the students, especially those from more traditional cultural backgrounds, may perceive a stigma associated with seeking psychological help [23]. Nevertheless, there are still some reported advantages with students utilising professional help-services, such as the ability to receive a clear diagnosis of one’s grieving pattern [24]. Seeking formal help may also provide the opportunity to find specific grief-focused therapies. Conversely, obtaining a diagnosis has been considered negative as it medicalises grief, which is a natural occurrence [24]. Interestingly, previous research conducted amongst adolescents tended to focus mainly on formal help-seeking behaviours. Parents have been noted to refer their children to formal help services, and adolescents seldom access these services on their own initiative [25]. However, research in university students generally does not report similar help-seeking patterns, as experiencing negative outcomes from bereavement does not necessarily equate to the utilisation of formal help-seeking resources [26].

Reasons for informal help-seeking in students include the ability to express grief without fear of judgement, feeling less isolated, and finding validation and normalisation of the grief experiences [24]. Whilst informal support methods are more popular in bereaved students, this has not translated into peers and family being well-equipped to providing support. Peers and family willing to provide support may be hindered by their expectation that grief is finite. Consequently, informal support for bereaved students may not be available for as long as they may need it [27]. The type of bereavement can also affect the quality of informal support received with those bereaved by suicide experiencing less informal help compared to other forms of bereavement [28]. Therefore, it is not uncommon for university students to describe feeling isolated from peers after experiencing a loss [29].

### 1.2. Grief Models

Contemporary models explaining the process of coping with grief and bereavement, such as the Dual Process Model of coping with bereavement (DPM) [30] and the Meaning Reconstruction and Loss Framework [31] may cater for both negative and positive outcomes of bereavement [18]. According to the DPM the grief process of a bereaved individual involves a process of oscillation between loss-oriented and restoration-oriented work. DPM was the first model to describe grief as a continuous process [30]. Previous approaches to understanding grief assumed bereaved individuals would gradually move away from pain associated with the loss, leading to its de-prioritisation in life [32]. However, grief can affect the lives of individuals in many ways and disassociating from the grief and loss completely cannot be assumed. DPM therefore revolutionised the understanding of grief, highlighting its continuous and dynamic process [33].

The Meaning of Reconstruction and Loss Framework (MRL) developed by Gillies and Neimeyer [34], describes the process undertaken by bereaved individuals in rebuilding their understanding of themselves and their environment [18]. Experiencing the bereavement, especially that of someone close, invites the bereaved person to reconsider their social environment [35]. Meaning reconstruction refers to processing the loss experienced and involves a shift from initially seeking an answer to “why” the death occurred to a focus on identifying and appreciating positive attributes in life [31].

According to the MRL framework, bereaved individuals will participate in three major activities, namely: sense-making, benefit-finding and identity change [34]. An individual’s “pre-loss meaning structures” will be remodelled when these three elements are applied, thereby creating new meaning structures encompassing elements of personal growth and new priorities in life [34]. Sense-making involves applying a new understanding to seek answers and reasoning behind the death, its associated burdens and the changes in an individual’s assumptions towards their social world [34,36]. Benefit-finding involves developing a new understanding of various meaning structures, acquired from forming new insights by way of interaction with the surrounding environment and through lessons learnt [34]. Whilst considered to have a positive effect overall, the acquired effects of this particular activity may not be demonstrated until sometime after the loss [37]. Finally, identity-change refers to adjustments in an individual’s self-perception and behavioural characteristics resulting from experiencing loss. Like benefit-finding, the changes experienced are generally considered positive, and embody elements of personal growth [34]. However, negative social and personality changes may also occur, impacting the drive to achieve life aspirations. This may also affect the quality of relationships with oneself and others, including with faith [38]. Within the MRL Framework, encountering stronger levels of distress are believed to interact positively with the amount of reconstruction undertaken [34].

### 1.3. Aims

Most studies in the field of grief and bereavement have been conducted with children/adolescents or adults [39,40,41], and little is known of how university students have experienced grief and personal growth. This study aimed to address this gap and intended to explore the detrimental effects of loss amongst university students, as well as how students may have experienced personal growth after the loss during this time in life. The study findings may inform clinicians and university services about the grief and personal growth in this population and may offer insights to service providers who offer information or treatment to bereaved students.

This study sought to answer the following research question: “How have university students experienced grief and/or personal growth after the death of a close person?”. To explore this question, three secondary questions were developed: (1) How have participants experienced their grief? (2) How have participants experienced help they might have received after the loss? (3) How have participants experienced any positive changes in their life after the loss? Although the research questions focus more on the positive attributes of personal growth, it is not merely synonymous with positive changes. As such, this study did not dismiss the possibility of negative attributes arising from personal growth.

## 2. Materials and Methods

### 2.1. Study Design and Sampling

This study was designed as a qualitative study according to the principles of Braun and Clarke [42] and involved thematic analysis of semi-structured interviews. The study adhered to the Consolidated Criteria for Reporting Qualitative Research (COREQ) [43]. Eligible participants were aged 18–30 years, currently enrolled as a student at an accredited Australian university and had experienced a bereavement after 12 years of age, inclusive. Potential participants were excluded if the bereavement concerned a non-human loss (e.g., death of a pet) or when the bereavement had occurred less than 6 months prior to participation in the study. We stratified the sample to include an equal proportion of male and female participants, participants who had been bereaved by different causes of death, and who were enrolled in different university faculties. Stratification was performed to ensure a variety of participants, rather than to conduct sub-group analysis. To minimise the risk of identification of participants, no other sociodemographic information was collected.

Recruitment took place between May and June 2020. It consisted of posting the study announcement on the online noticeboard accessible to all staff and students from The University of Melbourne (https://www.unimelb.edu.au/) (accessed on 16 February 2021), and on one of the researcher’s (J.T.) own Facebook page. The study announcements were publicly available and could be freely shared. It was decided to limit recruitment to a few online methods due to COVID-19 pandemic restrictions imposed by the Victorian State Government in early 2020. Participants were offered a $30.00 gift voucher as a reimbursement for their time. Based on the literature and our experience with qualitative research, we estimated that the study would require 12 to 15 participants to answer the research question [13,25,44,45].

In total, 66 students expressed interest in the study. All potential participants received a plain language statement (PLS) and a consent form detailing the collection of verbal consent at the beginning of the interview, and 45 potential participants responded positively. Reasons for not responding (*n* = 21) are unknown. Next, 14 participants were invited for an interview. The remaining participants were placed on a waiting list. The interview participants were selected based on the following characteristics: gender, the type of bereavement experienced, the relationship with the deceased, and university faculty. The participants’ ages ranged from 19 to 29 at the time of the interview (M = 23.4, SD = 3.98). The ages at the time of loss ranged from 13 to 27 (M = 19.9, SD = 4.62). Of the 14 participants, five participants had experienced the death of a grandparent, which was the most common form of bereavement. Four participants were bereaved by the death of an extended family member, and one participant had lost their father. The remaining four participants experienced the loss of a friend. The ages of the deceased individuals ranged from 16 to 94. Participants came from a range of faculties spanning both undergraduate and postgraduate studies at The University of Melbourne. The included faculties consisted of: Arts, Engineering, Education, Commerce and Medicine, Dentistry and Health Sciences. Across the participants, the causes of death encompassed both unexpected and expected deaths such as accidents, suicide, and short-term and long-term diseases.

### 2.2. Ethical Considerations

The Human Research Ethics Committee of The University of Melbourne approved the study (Ethics ID: 2056630.1). Before the start of the interview, participants had an opportunity to ask questions they may had about the study. The interviewer (J.T.) re-affirmed the voluntary nature of the study. Participants could skip interview questions, pause or withdraw from the interview at any point. All participants provided verbal consent before the start of the interview.

Originally, the interviews were to be conducted either in person or via Zoom/telephone. However, due to the COVID-19 pandemic we decided to conduct all interviews remotely (Zoom or telephone). Bearing in mind the potentially distressing nature of the interview matter, we prepared a participant risk protocol, and took care to ensure participants were as comfortable as possible during the interviews. When conducted on Zoom, participants were free to leave their camera off. The interview questions focused more on the positive experiences of the participants, following a chronological order of events until the present day. The interviews started and ended with warming up and cooling down questions, respectively. These included asking participants about their socio-demographic characteristics, academic background and about advice the participants may have for other bereaved students.

### 2.3. Data Collection

The study used a semi-structured interview format, with open-ended lead questions, which allowed us to focus on the personal growth aspects of grief and provided participants with an opportunity to elaborate on specific answers. The interview guide (Supplementary File 1: Interview guide) was created in consultation with the current literature (e.g., Balk et al. [29]), including literature on the DPM and MRL Framework [30,31,33,34,46,47]. Care was taken to change question wording to minimise bias towards the two models, and two mock interviews were conducted in advance with two individuals external to the study. One researcher (J.T.) conducted all the interviews. The second researcher (K.A.) monitored the mock interviews as well as the first two interviews conducted for the study.

Interviews were organised at a time suitable for the participants. The interviews were audio recorded and transcribed using the transcription service provided by NVivo 12 [48]. J.T. checked the transcripts for accuracy. To ensure participant confidentiality only de-identified data was used throughout the study.

### 2.4. Data Analysis

We conducted a thematic analysis involving a codebook created for this study. We based the process of coding and analysis on the principles of Braun and Clarke [42], which implied an iterative process of systematically identifying and organizing patterns of meaning (i.e., themes) in the data set. We used NVivo 12 for Mac (QSR International, Melbourne, Australia) [48] for coding and data management. Prior to the interviews, J.T. and K.K. generated deductive codes from the semi-structured interview questions. Following the interviews, J.T. and K.A. independently coded the same three transcripts to create inductive codes. Any disagreement was resolved by discussion. J.T. then coded the remaining transcripts. The codes were continuously reviewed and refined during the coding process. J.T. created a thematic map linking all codes together (Supplementary File 2: Thematic map). This visualization of the codes helped in the identification of themes in the data. J.T. and K.A. reviewed and refined the potential themes following the recommendations of Braun and Clarke [42]. To aid reflexivity, J.T., a graduating Master of Public Health student, undertook a series of related online modules offered by Mental Health First Aid Australia. K.A. is a social worker with extensive experience in the field of bereavement and mental health research. J.T. and K.A. held regular meetings to minimise researcher bias.

## 3. Results

The analysis identified four themes: (1) Sharing of grief as a coping mechanism, (2) Balance between grief reactions and moving forward in life, (3) Lessons learned and personal growth and (4) Adopting values from the deceased and continuing bonds. Table 1 summarizes the themes. Examples are provided for each theme; names are fictionalized and participant age is reported at the time of the interview.

### 3.1. Theme 1: Sharing of Grief as a Coping Mechanism

This theme comprised two aspects. The first aspect involved participants sharing their grief informally. Participants reported approaching friends or family to share their emotions, which was the more common help-seeking method found in this study. Participants emphasized that they felt comfortable in the presence of their family or friends and trusted them. For participants who experienced a familial loss, other family members were generally the first to be approached. Likewise, participants bereaved by the death of a friend tended to reach out towards other friends. Many participants cited the reason for this was due to the shared grief with either family or friends. They also stated that a degree of relatability was important in their decision of who was approached. Most participants felt that those who also knew the deceased person provided some comfort as they shared the same grief.

“*I think that a feeling of support that I was getting from the group of friends who experienced and went through the same thing as me, it was really soothing for me*”

(Teresa, aged 29; friend, accident).

Secondly, participants also engaged in formal help-seeking such as counselling, peer support and through social media. Participants accessed these services through university or school advertisements. Although less popular, participants still highlighted notable benefits from using psychological services and peer support groups, including having a chance to formally articulate their emotions, especially in dealing with the guilt and shock after an unexpected death. However, participants also noted negative experiences: the limited time of the psychological sessions often resulted in an abruptness in their ending.

“*I think sometimes it can be quite hard, if you’re digging up a lot of your family or personal or relationship traumas and then the session finishes and you kind of have to walk away, having regurgitated all of that. And she [i.e., psychologist] was quite conscious of trying to leave on a positive note, but I still think sometimes when you’ve brought certain things up, especially when they’re delving quite deep…you might trigger something you’ve not really thought about in a while*”

(Molly, aged 29; uncle, accident).

Participants who had not used formal help-services cited lack of awareness, difficult access, cultural stigma and the lack of personal relatability with the paid consultant as the main reasons. Similarly, some participants attempted to approach their peers but stated that they observed or experienced some discomfort and a lack of knowledge from peers when aiding them. Participants stated that they sought formal help services mainly when they considered that informal help was inadequate.

### 3.2. Theme 2: Balance between Coping with Grief and Moving Forward in Life

The grief reactions of the participants varied depending on the type of loss experienced, such as the cause of death and its expectedness. Participants commonly experienced initial feelings of shock and sadness. Those who were bereaved by a sudden death also struggled with feelings of guilt and regret. Initial grief reactions included feeling shocked, disbelief and immediately reaching out to other affected family members or friends with the intention of providing and receiving comfort.

As a means of positively reacting and coping with the grief, participants emphasised trying to focus on the future. Initially, this positive-thinking approach was undertaken either by themselves or with the help of specific very close friends or family. However, some participants stated that they deliberately withdrew from social interaction and preferred to process the grief by themselves. Participants felt that the extent of negative reactions such as shock, distress or regret, seemed to depend on the expectedness of the death, rather than on the type of bereavement. For most participants, the loss of the ‘expected deaths’ were of a familial nature, and most of the deceased were older adults.

“*Deep down in my heart, and I do want him to be around me for everyday life, but when I am awake, I just want to erase the bad memory. So, I think when I’m awake I don’t want to remember about him, but when I sleep, I want to remember him. I think that’s the reality of my heart that I want to remember more about him and cherish him. But when I’m awake, I just want to let it go*”

(Clare, aged 27; father, suicide).

Participants noted that over time, they had experienced a growing ability to come to terms with the loss, allowing continuation with their lives. However, several participants also continued experiencing grief. For some, this occurred periodically, for example, at formal ceremonies, at memorials or on social media.

“*I did feel sad, but I didn’t feel lost because I had already said goodbye. But then, like in the months after, I think back to all the nice memories and it makes me very sad. Like I’ve had some nice calls with my grandmother and stuff but it’s like it hit me more slowly. It wasn’t like something that made me sad when it happened. It’s more like, now living my life without him around is the thing that makes me sad*”

(Oscar, aged 22; grandfather, illness).

Overall, participants expressed the alternation between grieving and moving forward both positively and negatively. For example, the coping mechanisms of one participant included being directly involved in suicide awareness activities. As a result, the participant felt that the loss had a longer but positive impact on her life, as stated below.

“*I think the one thing that I’ve kind of taken from that time has been just being very active about suicide prevention. And I think like even at uni now, I’m involved in [activities] that involves spreading things like mental health awareness. And so, I think I’ve remained quite active in that area since then. That’s one of my sorts of main ways to remember him. [Suicide] has such widespread effects, it can be hard to see that…and I really would like, if I could do anything, just to keep that from happening to other people*”

(Kiara, aged 19; friend, suicide).

### 3.3. Theme 3: Lessons Learned and Personal Growth

Most participants underlined that they had grown as a person due to the loss. This was experienced in various ways, including a change in caring for others, perspective on life, views of themselves, discovering of new possibilities, and valuing others and relationships. Many participants stated that the lessons learnt resulted in social, physical and philosophical changes. For most participants, this was a positive experience.

Positive social changes included the new appreciation of relationships, changes in behaviour and an increased perception of closeness with others. Participants reflected upon their increased valuation of current relationships with both friends and family. They also highlighted an increase in care for others and displaying increased maturity.

“*I feel like I have grown up as a person. Like, I feel like I’m not the one that I used to be before all of these things happened to me. I’ve become more positive and not judgemental about something or people around me*”

(Ella, aged 25; friend’s mum, cancer).

However, this was not common to all participants. Some felt there was no change, and some noted having a more negative experience; for example, one participant mentioned being more wary of developing intimate relationships or seeking new friendships.

Philosophical changes included a change in views on mortality. Coupled with this, participants mentioned focusing on the positive aspects in their attitude towards life now. To help cope with the loss, participants talked about finding solace through practising religion and/or meditation.

Physical changes undertaken by some participants generally involved a change in location such as moving interstate or overseas. Participants deemed it as making the most of a new opportunity, and reported several benefits including establishing more independence, self-discovery and development of one’s strengths.

“*At the end, it’s the people that you’ve touched and left an impact on, the legacy that matters in the end. It has given me more perspective in the way that I deal with my own life choices and in the way that I probably also deal with the relationships that I have in my life. To have more appreciation for… to build personal relationships with others*”

(Hugo, aged 29; grandmother, cancer).

### 3.4. Theme 4: Adopting Values from the Deceased and Continuing Bonds

Most of the losses experienced were from individuals who played a substantial role in the participant’s life. Participants generally stated they had a close relationship with the deceased person. When the loss was of someone older than the participant, they generally mentioned the deceased having memorable values, traits and life experiences. Examples included determination and fearlessness. Participants were grateful for the sound advice that they had received from the deceased, and they highlighted values adopted from the deceased person as being useful in many aspects of life.

“*I think you can say the principles of- that like she lived… she struggled and managed to do a really good job. So, if something is a bit tough, I think I can pull it together to get it done…I think just being empathetic towards people that come from a shittier background than you and not assuming that everyone had the same luxuries. Don’t look down on people just for being poor where they come from*”

(Mark, aged 25; grandmother, cancer).

For participants where the deceased was closer in age, memorable attributes focused more on the personality traits of the deceased. Participants stated being inspired to adopt similar approaches in life. When the loss had occurred during their time in high school, participants talked about being inspired to work hard academically. Of note, two participants stated that they had not experienced major changes in their lives after the loss. However, they felt that their relationship with the deceased was not very close.

Some participants performed rituals as a means of continuing bonds. For example, participants included the deceased person in their prayers, visited the social media profile or the grave, or talked about the deceased with others. Participants mentioned using this time to reflect upon the relationship they had with the deceased person and to cherish memories.

“*I think like two or three times a year, I commented on his last post. Like ‘Happy New Year [friend’s name], I’m sure you are having more fun da-da’. Or for example, ‘happy happy…like some holiday’. And there are hundreds and hundreds of comments on his last post from his sister or his friends and I think… Yeah I don’t know if it’s healthy or not, but yeah, I commented on his last post that I think he is still a dear friend of mine who I don’t have in this life, but I have his memories in my mind*”

(Hannah, aged 29; friend, accident).

## 4. Discussion

Experiencing the death of someone close potentially impacts an individual’s life negatively and/or positively. Implications of the loss can span across the bereaved person’s social and personal outlook on life. This study aimed to explore the experiences of bereavement amongst emerging adults in a university setting, with a specific focus on personal growth in this population. The sharing of grief as a coping mechanism by many of the study participants is supported by both the DPM and MRL Framework [30,34]. Participants in this study found it beneficial in their grieving process, which is in line with studies demonstrating that sharing may contribute to personal growth [14]. Specifically, all participants referred to at least one or more of the five domains of personal growth, with increased self-perception mentioned most commonly [12].

The participants considered relatability in sharing grief was important; especially in seeking informal help. Previous studies conducted with adolescents and adults also reported on the importance of the connection and understanding between the person sharing grief and the listener [25,49]. As such, there may be a similarity between university students and adolescents in both help-seeking and help-giving behaviour. This was especially notable when participants spoke about bereavement that occurred during high school. However, it seems that university students also demonstrated independence and self-reliance in their grief reactions, possibly more compared to adolescents. This supports findings attributing grief reactions to the more independent environment of university [1,3]. Further studies involving both adolescents and university students may shed light on similarities or differences in grief reactions between both groups.

Amongst the study participants, informal support was the most popular form of help-seeking. The preference for informal help is similar to adolescent studies where the preference for seeking peer support over professionals increases with age [25,50]. However, the findings in our study also contradict a claim by Howard-Sharp et al. [50] that peer support can adequately supplement familial support, as peer support seemed to be of variable quality according to our participants. Interestingly, study participants who utilised formal help only turned towards this method when peer help was considered unavailable or inadequate. The findings indicate the complementary roles of informal and formal support for bereaved university students.

Study participants highlighted several barriers to formal support. In line with previous research, participants in this study reported on deterrents such as cultural stigma [23], a lack of awareness of the services, distrust in the health service and belief that independent grieving was adequate [25,51,52]. However, others emphasized the lack of success they had in accessing the university help services. Our participants cited inadequate supply of services as the primary difficulty encountered, with availability difficult to come by. This is unlike other studies in both adolescents and university students focusing on the quality of formal help services and the experiences of using them [51,52,53,54]. The barriers found in this study strongly suggest that there is also an issue with the quantity available, not just the quality of service and the user experience.

Although participants reported an increase in self-perception, they did not necessarily feel that this resulted in a marked identity change as suggested in both the DPM and MRL Framework [30,31,33,34]. ‘Identity-change’ constitutes a key activity performed in the process of personal growth, subsequently resulting in identity reconstruction after experiencing the loss [34,46]. However, the extent of the ‘identity change’ aspect may directly correlate with the age of the individual at the time of the loss [55,56]. For example, in experiencing a grandparent’s death, there may be another, older individual to step in and perform more of the ‘caretaker’ role in handling grief-related matters. Participants who had experienced peer, sibling or parental bereavement, seemed to reflect more on identity change. This may be due to the bereaved individual being more involved in the aftermath of the loss. For example, they may have to step into a role with more responsibilities, supporting others also directly impacted [3].

Similarly, the types of value adopted by the participants seem to be influenced by the age of the deceased. This is in line with the literature stating that older individuals have more life lessons to give compared to younger [56]. Many participants also reported feeling there was a philosophical change that occurred due to their relationship with the person they had lost. However, regarding development of personal growth, there may be other factors to consider. Tertiary education is flexible in its occurrence during an individual’s life, so previous life experiences such as those gained through work may play a role. Additionally, the unique environment of university itself presents the opportunity to develop oneself and challenges social understanding. Hence, its influence in this transitional time should also be accounted for [3,57]. It also begs the question for future research of how the results may differ in this age group external to university, where there may be more variation in life experiences.

Several participants reported developing philosophical changes such as changes in perspective on life when they turned towards religion to find solace. These participants believed that religion provided a sense of stability and support during the distressing grieving period. Previous research has also demonstrated that faith may contribute to making sense of the death and encourage personal growth [58,59,60]. Amongst our study participants, a variety of religious backgrounds was present, and the differences in understanding death may offer a myriad of life lessons [61]. Existential changes experienced by the participants are also supported by the literature linking religion and its influence on moral reasoning [60].

Interestingly, non-religious participants also reported feeling philosophical changes, suggesting that religion is not necessary for stimulating personal growth. This is supported by literature indicating that an individual’s characteristics and preconceived notion of life prior to the loss is the key factor for meaning-making, benefit-finding and potential identity change [31,62]. Our study also supports findings from Smith-Stoner [63] suggesting that the extent in changing methods of existential meaning-making are one of the more influential factors for philosophical changes. As a result, our findings suggest that spiritual and/or existential changes may occur in religious and non-religious students, as both participants with and without a religion mentioned philosophical changes. Further research may clarify if the moral elements in religion or other social structures may contribute to this type of personal growth in bereaved students.

### 4.1. Limitations

This study was not without limitations. Due to the COVID-19 pandemic restrictions implemented in the state of Victoria, and specifically in Melbourne, Australia, recruitment for the study had to be limited to online methods. As a result, all participants were students from The University of Melbourne. However, from the onset it was expected that most participants would be from this university as the main recruitment was conducted through its online noticeboard. Future studies may include students from different universities. Given the limited time available to conduct the study (i.e., one academic year), the recruitment period was limited to 20 days. However, the study immediately received a high number of expressions of interest, and we succeeded in recruiting a sufficiently large and variated sample, resulting in a rich data set. Still, the study did not include a formal assessment of data saturation and future studies may include larger samples. The study relied on self-report of experiences which have occurred at various times in participants’ lives, which is open to recall bias. Additionally, the study did not utilise a formal quantitative measurement tool. Therefore, a complimentary study may be conducted to further validate findings of this study. Despite the variation in the sample, not all types of relationship were represented. For example, no participant had experienced the loss of a younger friend or family member. As a result, the study findings may have limited generalisability to university students who have experienced this form of bereavement.

### 4.2. Implications

The findings strongly suggest that universities must provide services and resources that are appropriate and accessible to students, enabling them to console bereaved peers. Having a wide range of easily accessible materials catering to students may take pressure off the professional services. Further, importance should be placed on the ability to share grief with friends and family. Hence, clinicians working with bereaved students may focus on their relationships with peers and family to further facilitate informal support for the bereaved student. Finally, the study revealed a clear need for further research on facilitating informal and formal help-seeking in bereaved students and service delivery for those who may need professional help.

## 5. Conclusions

This study explored how university students experienced grief and personal growth, and the findings emphasize the impact bereavement can have on their life. University constitutes a new environment for emerging adults requiring psychosocial independence and maturity, which is reflected in their grief reactions. There appears to be a preference towards informal help-seeking through sharing grief with peers or family. Participants oscillated between grieving and moving forwards in life and they recounted lessons learnt from the bereavement. Personal growth was mostly experienced through changes to self-perception and philosophical attitudes towards life. Values adopted seemingly related to the age of the deceased and of the bereaved person at the time of loss. Moreover, university represents a time of self-discovery in an emerging adults’ life, adding to the potential range of personal growth undertaken.

Our findings emphasize the importance of universities providing widely available and appropriate resources to students. To better assist bereaved university students, the material should be catered to peers and/or family. However, in the absence of informal help, professional resources should also be available, and clinicians should focus on friends and family to facilitate support for the bereaved student. Further research may focus on encouraging formal and informal help-seeking in bereaved students and service delivery to those who need professional help.

## Figures and Tables

**Table 1:** Summary of themes.

Theme	Content
1. Sharing of grief as a coping mechanism	Formal help-seeking methods Informal help-seeking methods
2. Balance between coping with grief and moving forward in life	Reactions from expected deaths Reactions from unexpected deaths Method of coming to terms with the loss
3. Lessons learned and personal growth	Positive attributes acquired from experiencing the loss Negative attributes acquired from experiencing the loss
4. Adopting values from the deceased and continuing bonds	Incorporating aspects from the deceased into life nowadays Type of relationship between the deceased and the bereaved

## Data Availability

Data sharing is not applicable to this article.

## Supplementary Materials

The following are available online at https://www.mdpi.com/1660-4601/18/4/1899/s1, Supplementary File 1: Interview guide; Supplementary File 2: Thematic map.
