# Supplementary material for: The Experiences of Grief and Personal Growth in University Students: A Qualitative Study
Source: Int J Environ Res Public Health. 2021 Feb 16;18(4):1899. doi: 10.3390/ijerph18041899 (PMC7920249; doi:10.3390/ijerph18041899)

International Journal of
Environmental Research and Public Health

MDPI

Project: The Experiences of Grief and Personal Growth in Bereaved University Students

Jovita Tan – Student Researcher, Master of Public Health student, Melbourne School of Population and Global Health, The University of Melbourne, Email: jovitat@student.unimelb.edu.au

Dr Karl Andriessen – Supervisor, Centre for Mental Health, Melbourne School of Population and Global Health, The University of Melbourne, Email: karl.andriessen@unimelb.edu.au

Supplementary File 1. Interview guide, semi-structured interviews.

Introduction

The interviewer will introduce themselves, welcome the participant, and socialise so that participants feel at ease. The interviewer will not start the interview abruptly but will reiterate the purpose and the context of the interview, and participants will have the opportunity to ask any questions they still might have about the study. Participants will be reminded that participation is voluntary and that they can pause or withdraw at any time.

Verbal consent

The interviewer will ask the following questions:

a) Have you had the opportunity to read the Plain Language Statement? Have you any questions about the document, including the items of consent listed in the appendix of the document?
b) Do you understand the purpose of the study?
c) Do you understand that participation is voluntary and that you are free to withdraw anytime without explanation or prejudice?
d) Do you understand that we will treat the information that you provide as confidential, that the data will be stored on a password protected computer, and that you will not be identifiable in any publication from this study.
e) Do you understand that the interview will be audio-recorded?
f) Do you consent to take part in the study?

About yourself

a) How old are you now?
b) What gender do you identify as?

Could you tell me a bit about the person who has died? (If the interviewee has lost more than one person, the interview will focus on the loss that was most important for the interviewee).

a. Who was the person who has died? What was your relationship with them? (prompt: for example, father, mother, brother, sister, friend, school mate, neighbour)
b. How old were you at the time? How long ago was this?
c. How old was the deceased when they died?
d. How did the person die? What was the cause of death?
e. How sudden, expected or unexpected was the death?
f. How did you feel or react at the time of death? (prompt: What were your thoughts or what did you do after the loss?)

Have you received any help after the loss?

a) If so: What kind of help? (e.g., emotional, practical, …)
i. From whom?

ii. Formal help from psychologist, counsellor, psychiatrist, peer support group, helplines, online, social media
iii. Informal help from family or friends
b) Was the help the you received helpful in any way? (prompt: Why or how was it helpful? What made it helpful or unhelpful?)

How important is the deceased person for you now?

a) Do you still feel connected with this person? Is the person still present in your life?
b) If so, how is this connection or presence in your life? (prompt: through memories, souvenirs, places, rituals, as a role model…)
c) Is this something ongoing, or more related to specific dates or events? (prompt: do you always feel the connection, is it always there, or is it something that comes and goes?
d) Is it something that you can control, or is it more something that happens to you?
e) How do you feel about this presence? What does it mean to you? (prompt: Do you feel that this supports you in any way, or is it more something that makes you feel uneasy?)

Looking back, since the loss, do you feel that the loss has changed your life? Have you learned anything from the whole experience?

a) Do you think there has there been any change in your outlook on life? (prompt: Do you feel that some things have become more important to you compared to before?)
b) Do you feel any changes in your strengths as a person? (prompt: do you feel any changes, for example, regarding self-reliance or confidence?)
c) Do you experience any changes in your relationships with family or friends? (prompt: do you feel any difference regarding feeling empathy or compassion for others?)
d) Have you found new possibilities, interests, pathways in your life? (prompt: for example, a new field of study, or a career switch).
e) Have you experienced any spiritual or existential changes? (prompt: have you experienced any changes regarding how you perceive mortality, or feeling connected with humanity or spiritual aspects of life?)

What has contributed to these changes? What has brought you there? (prompt: the help that you received after the loss? Your coping mechanisms? Feeling connected with the deceased person?)

How do you perceive these changes? (prompt, for example, as something positive, enriching, or as a burden? In what way?)

Is there any advice that you would like to give to other young persons who have lost someone important in their life?

Is there anything that you would like to add? Something that is important for you and that we have not discussed?

Concluding the interview

The interviewer will thank the participant, reiterate the importance of their contribution to the study, and how the information will be processed. The interviewer will remind participants that they can contact the service providers mentioned in the study documents

Supplementary File 2. Thematic map.

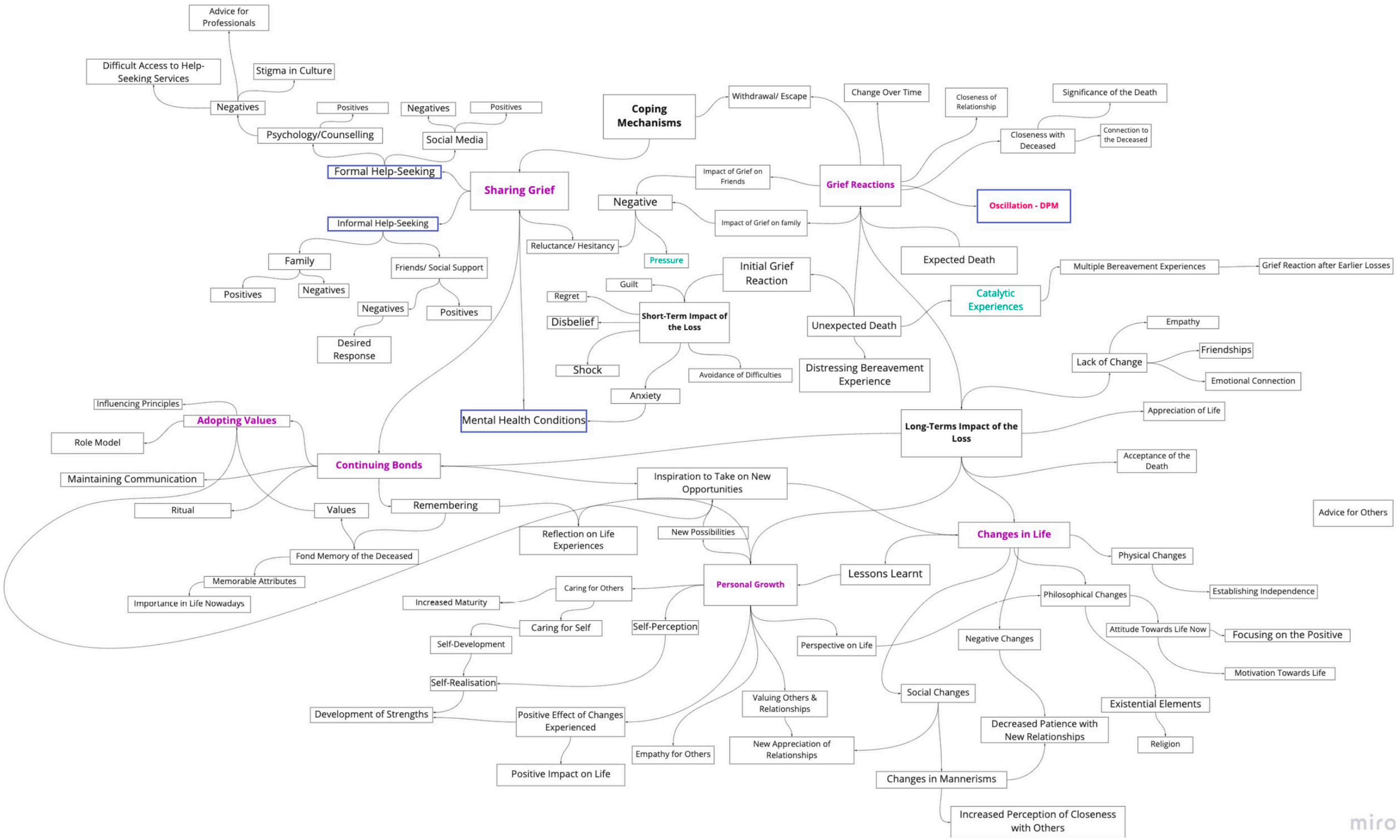

Supplement: Supplementary file 1 [file ijerph-18-01899-s001.pdf]
